# Supplementary material for: Exploring the mechanisms by which camel lactoferrin can kill Salmonella enterica serovar typhimurium and Shigella sonnei
Source: PeerJ. 2023 Jan 30;11:e14809. doi: 10.7717/peerj.14809 (PMC9893911; doi:10.7717/peerj.14809)
Supplement: Supplemental Information 2 [file peerj-11-14809-s002.docx]

**Supplementary Materials**

**Exploring the mechanisms by which camel lactoferrin can kill *Salmonella enterica* serovar *typhimurium* and *Shigella sonnei***

Hussein A. Almehdar^1^, Nawal Abd El-Baky^2^, Ehab H. Mattar^1^, Raed S. Albiheyri^1^, Atif A. Bamagoos^1^, Vladimir N. Uversky ^1,3,*^, and Elrashdy M. Redwan ^1,2,*^

*^1^Department of Biological Sciences, Faculty of Sciences, King Abdulaziz University, P.O. Box 80203, Jeddah, Saudi Arabia.*

*^2^Therapeutic and Protective Proteins Laboratory, Protein Research Department, Genetic Engineering and Biotechnology Research Institute, City of Scientific Research and Technological Applications, New Borg EL-Arab, 21934, Alexandria, Egypt.*

*^3^Department of Molecular Medicine and USF Health Byrd Alzheimer's Research Institute, Morsani College of Medicine, University of South Florida, Tampa, FL, USA.*

^*^**Corresponding author**:

E.M. Redwan: [lradwan@kau.edu.sa](mailto:lradwan@kau.edu.sa)

**Table S1.** Antibacterial effects of different lactoferrins on *S. typhimurium* and *S. sonnei* growth after 1, 3, 6, 12, and 24 h of incubation.

| **Lf** | **Concentration**  **(mg/ml)** | **OD at 620 nm (mean ± SEM)** | | | | | | | | | | | | | |
| --- | --- | --- | --- | --- | --- | --- | --- | --- | --- | --- | --- | --- | --- | --- | --- |
|  |  | ***S. typhimurium*** | | | | | | | | ***S. sonnei*** | | | | | |
|  |  | **0 h** | | | **1 h** | **3 h** | **6 h** | **12 h** | **24 h** | **0 h** | **1 h** | **3 h** | **6 h** | **12 h** | **24 h** |
| cLf1 | 0 | 0.003  ±0.002 | | | 0.294  ±0.01 | 0.451  ±0.011 | 0.919  ±0.005 | 2.558  ±0.036 | 2.901  ±0.023 | 0.004  ±0.002 | 0.323  ±0.019 | 0.491  ±0.01 | 0.881  ±0.005 | 2.311  ±0.007 | 3.107  ±0.005 |
|  | 3 | 0.005  ±0.002 | 0.207  ±0.013 | | | CIG | CIG | CIG | CIG | 0.007  ±0.003 | 0.259  ±0.01 | CIG | CIG | CIG | CIG |
|  | 2.5 | 0.004  ±0.001 | 0.287  ±0.017 | | | CIG | CIG | CIG | CIG | 0.005  ±0.001 | 0.291  ±0.012 | CIG | CIG | CIG | CIG |
|  | 2 | 0.005  ±0.001 | 0.265  ±0.011 | | | CIG | CIG | CIG | CIG | 0.003  ±0.002 | 0.277  ±0.009 | CIG | CIG | CIG | CIG |
|  | 1.5 | 0.004  ±0.002 | 0.295  ±0.005 | | | CIG | CIG | CIG | CIG | 0.005  ±0.002 | 0.305  ±0.009 | CIG | CIG | CIG | CIG |
|  | 1 | 0.003  ±0.002 | 0.187  ±0.008 | | | CIG | CIG | CIG | CIG | 0.008  ±0.003 | 0.307  ±0.013 | CIG | CIG | CIG | CIG |
|  | 0.75 | 0.002  ±0.001 | 0.281  ±0.003 | | | 0.122  ±0.003 | CIG | CIG | CIG | 0.004  ±0.001 | 0.317  ±0.01 | 0.152  ±0.007 | CIG | CIG | CIG |
|  | 0.5 | 0.005  ±0.002 | | 0.274  ±0.003 | | 0.174  ±0.004 | 0.082  ±0.002 | CIG | CIG | 0.003  ±0.002 | 0.329  ±0.005 | 0.191  ±0.01 | 0.100  ±0.004 | CIG | CIG |
|  | 0.25 | 0.005  ±0.002 | | 0.187  ±0.003 | | 0.106  ±0.004 | 0.092  ±0.002 | 0.077  ±0.002 | CIG | 0.003  ±0.001 | 0.210  ±0.001 | 0.155  ±0.005 | 0.119  ±0.004 | 0.09  ±0.006 | CIG |
|  | 0.125 | 0.005  ±0.002 | | 0.374  ±0.003 | | 0.221  ±0.002 | 0.145  ±0.003 | 0.071  ±0.003 | CIG | 0.005  ±0.001 | 0.279  ±0.005 | 0.183  ±0.005 | 0.117  ±0.004 | 0.094  ±0.002 | CIG |
| cLf2 | 0 | 0.003  ±0.002 | | 0.351  ±0.011 | | 0.492  ±0.017 | 0.879  ±0.003 | 2.481  ±0.024 | 2.773  ±0.031 | 0.004  ±0.002 | 0.402  ±0.013 | 0.604  ±0.011 | 0.958  ±0.005 | 2.378  ±0.009 | 3.217  ±0.012 |
|  | 3 | 0.005  ±0.001 | | 0.315  ±0.009 | | CIG | CIG | CIG | CIG | 0.004  ±0.001 | 0.271  ±0.007 | CIG | CIG | CIG | CIG |
|  | 2.5 | 0.002  ±0.001 | | 0.216  ±0.004 | | CIG | CIG | CIG | CIG | 0.005  ±0.001 | 0.243  ±0.006 | CIG | CIG | CIG | CIG |
|  | 2 | 0.007  ±0.001 | | 0.268  ±0.007 | | CIG | CIG | CIG | CIG | 0.005  ±0.002 | 0.224  ±0.01 | CIG | CIG | CIG | CIG |
|  | 1.5 | 0.004  ±0.002 | | 0.226  ±0.002 | | CIG | CIG | CIG | CIG | 0.005  ±0.003 | 0.197  ±0.009 | CIG | CIG | CIG | CIG |
|  | 1 | 0.004  ±0.002 | | 0.291  ±0.006 | | CIG | CIG | CIG | CIG | 0.007  ±0.002 | 0.319  ±0.01 | CIG | CIG | CIG | CIG |
|  | 0.75 | 0.003  ±0.001 | | 0.288  ±0.002 | | 0.135  ±0.002 | CIG | CIG | CIG | 0.005  ±0.003 | 0.320  ±0.007 | 0.181  ±0.003 | CIG | CIG | CIG |
|  | 0.5 | 0.005  ±0.001 | | 0.379  ±0.005 | | 0.243  ±0.004 | 0.171  ±0.002 | CIG | CIG | 0.003  ±0.001 | 0.302  ±0.01 | 0.192  ±0.002 | 0.115  ±0.009 | CIG | CIG |
|  | 0.25 | 0.003  ±0.002 | | 0.300  ±0.002 | | 0.211  ±0.006 | 0.142  ±0.004 | 0.083  ±0.003 | CIG | 0.005  ±0.001 | 0.279  ±0.005 | 0.192  ±0.004 | 0.125  ±0.009 | 0.08  ±0.004 | CIG |
|  | 0.125 | 0.005  ±0.001 | | 0.279  ±0.01 | | 0.144  ±0.003 | 0.095  ±0.004 | 0.062  ±0.002 | CIG | 0.007  ±0.001 | 0.285  ±0.005 | 0.138  ±0.005 | 0.09  ±0.001 | 0.075  ±0.006 | CIG |
| cLf3 | 0 | 0.003  ±0.002 | | 0.258  ±0.009 | | 0.485  ±0.021 | 0.823  ±0.015 | 2.377  ±0.023 | 2.675  ±0.033 | 0.003  ±0.002 | 0.355  ±0.011 | 0.541  ±0.016 | 0.917  ±0.008 | 2.506  ±0.015 | 3.416  ±0.027 |
|  | 3 | 0.005  ±0.001 | | 0.285  ±0.009 | | CIG | CIG | CIG | CIG | 0.004  ±0.002 | 0.325  ±0.01 | CIG | CIG | CIG | CIG |
|  | 2.5 | 0.002  ±0.001 | | 0.236  ±0.014 | | CIG | CIG | CIG | CIG | 0.003  ±0.001 | 0.271  ±0.009 | CIG | CIG | CIG | CIG |
|  | 2 | 0.005  ±0.001 | | 0.288  ±0.011 | | CIG | CIG | CIG | CIG | 0.007  ±0.001 | 0.196  ±0.007 | CIG | CIG | CIG | CIG |
|  | 1.5 | 0.002  ±0.002 | | 0.316  ±0.007 | | CIG | CIG | CIG | CIG | 0.004  ±0.002 | 0.270  ±0.002 | CIG | CIG | CIG | CIG |
|  | 1 | 0.005  ±0.002 | | 0.221  ±0.005 | | CIG | CIG | CIG | CIG | 0.004  ±0.002 | 0.191  ±0.006 | CIG | CIG | CIG | CIG |
|  | 0.75 | 0.003  ±0.002 | | 0.295  ±0.005 | | 0.174  ±0.007 | CIG | CIG | CIG | 0.003  ±0.001 | 0.308  ±0.002 | 0.123  ±0.003 | CIG | CIG | CIG |
|  | 0.5 | 0.004  ±0.001 | | 0.348  ±0.011 | | 0.212  ±0.009 | 0.124  ±0.003 | CIG | CIG | 0.005  ±0.001 | 0.261  ±0.005 | 0.193  ±0.004 | 0.102  ±0.002 | CIG | CIG |
|  | 0.25 | 0.003  ±0.001 | | 0.370  ±0.009 | | 0.276  ±0.002 | 0.199  ±0.005 | 0.100  ±0.003 | CIG | 0.005  ±0.002 | 0.388  ±0.01 | 0.248  ±0.005 | 0.125  ±0.002 | 0.084  ±0.001 | CIG |
|  | 0.125 | 0.005  ±0.001 | | 0.297  ±0.005 | | 0.214  ±0.004 | 0.145  ±0.002 | 0.100  ±0.002 | CIG | 0.004  ±0.001 | 0.336  ±0.01 | 0.194  ±0.006 | 0.132  ±0.004 | 0.093  ±0.003 | CIG |
| cLf4 | 0 | 0.003  ±0.002 | | 0.299  ±0.01 | | 0.477  ±0.009 | 0.754  ±0.005 | 2.465  ±0.016 | 2.891  ±0.013 | 0.004  ±0.002 | 0.302  ±0.008 | 0.435  ±0.011 | 0.788  ±0.007 | 2.505  ±0.012 | 2.771  ±0.017 |
|  | 3 | 0.005  ±0.002 | | 0.364  ±0.013 | | CIG | CIG | CIG | CIG | 0.004  ±0.002 | 0.300  ±0.01 | CIG | CIG | CIG | CIG |
|  | 2.5 | 0.004  ±0.001 | | 0.244  ±0.012 | | CIG | CIG | CIG | CIG | 0.003  ±0.001 | 0.277  ±0.015 | CIG | CIG | CIG | CIG |
|  | 2 | 0.005  ±0.001 | | 0.175  ±0.013 | | CIG | CIG | CIG | CIG | 0.003  ±0.001 | 0.183  ±0.017 | CIG | CIG | CIG | CIG |
|  | 1.5 | 0.004  ±0.002 | | 0.195  ±0.007 | | CIG | CIG | CIG | CIG | 0.004  ±0.002 | 0.217  ±0.004 | CIG | CIG | CIG | CIG |
|  | 1 | 0.003  ±0.001 | | 0.237  ±0.007 | | CIG | CIG | CIG | CIG | 0.005  ±0.001 | 0.230  ±0.009 | CIG | CIG | CIG | CIG |
|  | 0.75 | 0.002  ±0.001 | | 0.177  ±0.005 | | 0.091  ±0.004 | CIG | CIG | CIG | 0.004  ±0.001 | 0.244  ±0.002 | 0.117  ±0.009 | CIG | CIG | CIG |
|  | 0.5 | 0.005  ±0.002 | | 0.298  ±0.006 | | 0.200  ±0.007 | 0.161  ±0.006 | CIG | CIG | 0.005  ±0.002 | 0.247  ±0.004 | 0.195  ±0.005 | 0.096  ±0.002 | CIG | CIG |
|  | 0.25 | 0.005  ±0.002 | | 0.336  ±0.005 | | 0.264  ±0.007 | 0.121  ±0.005 | 0.09  ±0.001 | CIG | 0.005  ±0.002 | 0.327  ±0.004 | 0.200  ±0.01 | 0.155  ±0.007 | 0.088  ±0.004 | CIG |
|  | 0.125 | 0.005  ±0.001 | | 0.317  ±0.005 | | 0.211  ±0.004 | 0.09 ±0.002 | 0.07  ±0.003 | CIG | 0.005  ±0.001 | 0.277  ±0.003 | 0.180  ±0.008 | 0.097 ±0.001 | 0.081  ±0.004 | CIG |
| hLf | 0 | 0.003  ±0.002 | | 0.431  ±0.01 | | 0.599  ±0.013 | 0.954  ±0.005 | 2.266  ±0.014 | 2.781  ±0.021 | 0.005  ±0.002 | 0.390  ±0.009 | 0.604  ±0.015 | 1.022  ±0.027 | 2.320  ±0.01 | 2.905  ±0.025 |
|  | 3 | 0.005  ±0.001 | | 0.385  ±0.009 | | CIG | CIG | CIG | CIG | 0.004  ±0.001 | 0.310  ±0.018 | CIG | CIG | CIG | CIG |
|  | 2.5 | 0.002  ±0.001 | | 0.206  ±0.007 | | CIG | CIG | CIG | CIG | 0.004  ±0.003 | 0.254  ±0.009 | CIG | CIG | CIG | CIG |
|  | 2 | 0.007  ±0.001 | | 0.168  ±0.007 | | CIG | CIG | CIG | CIG | 0.007  ±0.001 | 0.173  ±0.01 | CIG | CIG | CIG | CIG |
|  | 1.5 | 0.004  ±0.002 | | 0.176  ±0.002 | | CIG | CIG | CIG | CIG | 0.005  ±0.002 | 0.184  ±0.005 | 0.107  ±0.009 | CIG | CIG | CIG |
|  | 1 | 0.004  ±0.002 | | 0.201  ±0.01 | | 0.117  ±0.006 | CIG | CIG | CIG | 0.003  ±0.002 | 0.280  ±0.017 | 0.166  ±0.016 | 0.086  ±0.007 | CIG | CIG |
|  | 0.75 | 0.003  ±0.001 | | 0.273  ±0.002 | | 0.155  ±0.004 | 0.123  ±0.003 | CIG | CIG | 0.005  ±0.001 | 0.329  ±0.008 | 0.619  ±0.016 | 1.260  ±0.013 | 1.637  ±0.025 | 2.760  ±0.016 |
|  | 0.5 | 0.005  ±0.001 | | 0.279  ±0.013 | | 0.687  ±0.01 | 1.181  ±0.006 | 1.570  ±0.007 | 2.567  ±0.022 | 0.007  ±0.001 | 0.349  ±0.008 | 0.728  ±0.019 | 1.390  ±0.016 | 2.056  ±0.022 | 2.941  ±0.017 |
|  | 0.25 | 0.003  ±0.001 | | 0.273  ±0.008 | | 0.597  ±0.007 | 1.177  ±0.004 | 2.566  ±0.011 | 3.066  ±0.014 | 0.005  ±0.002 | 0.384  ±0.011 | 0.735  ±0.012 | 1.284  ±0.017 | 2.118  ±0.009 | 2.970  ±0.011 |
|  | 0.125 | 0.005  ±0.001 | | 0.379  ±0.013 | | 0.694  ±0.011 | 1.564  ±0.016 | 2.334  ±0.021 | 2.982  ±0.013 | 0.007  ±0.001 | 0.244  ±0.003 | 0.677  ±0.009 | 1.855  ±0.011 | 2.638  ±0.024 | 3.082  ±0.009 |
| bLf | 0 | 0.003  ±0.002 | | 0.451  ±0.014 | | 0.684  ±0.024 | 1.699  ±0.035 | 2.676  ±0.022 | 2.911  ±0.014 | 0.004  ±0.002 | 0.490  ±0.014 | 0.743  ±0.02 | 1.422  ±0.007 | 2.013  ±0.02 | 2.800  ±0.01 |
|  | 3 | 0.005  ±0.001 | | 0.375  ±0.011 | | CIG | CIG | CIG | CIG | 0.005  ±0.001 | 0.328  ±0.007 | CIG | CIG | CIG | CIG |
|  | 2.5 | 0.002  ±0.001 | | 0.214  ±0.008 | | CIG | CIG | CIG | CIG | 0.004  ±0.001 | 0.278  ±0.01 | CIG | CIG | CIG | CIG |
|  | 2 | 0.007  ±0.001 | | 0.258  ±0.007 | | CIG | CIG | CIG | CIG | 0.005  ±0.001 | 0.299  ±0.017 | CIG | CIG | CIG | CIG |
|  | 1.5 | 0.004  ±0.002 | | 0.233  ±0.002 | | CIG | CIG | CIG | CIG | 0.003  ±0.002 | 0.258  ±0.01 | 0.138  ±0.009 | CIG | CIG | CIG |
|  | 1 | 0.004  ±0.002 | | 0.344  ±0.006 | | 0.208  ±0.003 | CIG | CIG | CIG | 0.005  ±0.002 | 0.329  ±0.011 | 0.261  ±0.014 | 0.156  ±0.004 | CIG | CIG |
|  | 0.75 | 0.003  ±0.001 | | 0.388  ±0.002 | | 0.219  ±0.002 | 0.158  ±0.006 | CIG | CIG | 0.005  ±0.001 | 0.287  ±0.015 | 0.594  ±0.018 | 0.946  ±0.012 | 1.397  ±0.023 | 2.300  ±0.01 |
|  | 0.5 | 0.005  ±0.001 | | 0.297  ±0.005 | | 0.640  ±0.004 | 1.126  ±0.002 | 1.519  ±0.013 | 2.335  ±0.007 | 0.004  ±0.001 | 0.283  ±0.01 | 0.624  ±0.01 | 0.983  ±0.017 | 1.475  ±0.02 | 2.544  ±0.019 |
|  | 0.25 | 0.003  ±0.001 | | 0.275  ±0.002 | | 0.587  ±0.01 | 1.181  ±0.022 | 1.499  ±0.007 | 2.576  ±0.002 | 0.005  ±0.001 | 0.296  ±0.004 | 0.530  ±0.007 | 1.029  ±0.013 | 1.622  ±0.017 | 2.700  ±0.01 |
|  | 0.125 | 0.005  ±0.001 | | 0.239  ±0.005 | | 0.644  ±0.003 | 1.566  ±0.014 | 2.377  ±0.007 | 2.865  ±0.006 | 0.003  ±0.002 | 0.288  ±0.007 | 0.570  ±0.011 | 1.400  ±0.009 | 2.624  ±0.015 | 3.012  ±0.021 |

*Abbreviations:* CIG, Complete inhibition of growth (OD ≤ 0.05).

**
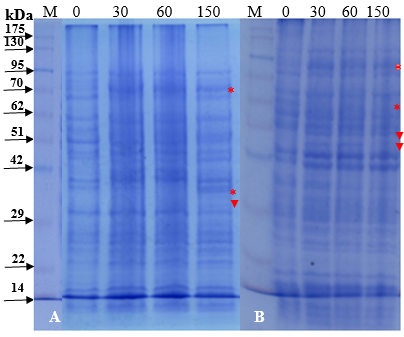
**

**Figure S1**. 12.5% reducing SDS-PAGE for camel lactoferrin effect on protein profile of *S. typhimurium* **(A)** and *S. sonnei* **(B)** at 0, 30, 60, and 150 min. The kinetics of changes in bacterial proteins were followed through sequential chronological SDS-PAGE profiling. The content of bacterial cells was separated on 12.5% SDS-PAGE after boiling for 7 min, stained with Coomassie brilliant blue and destained. Panels A and B represent individual gels, which were not modified in any way.

**
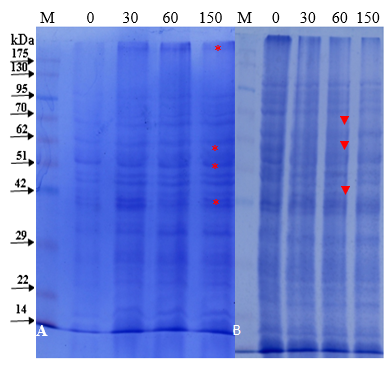
**

**Figure S2**. 12.5% reducing SDS-PAGE for carbenicillin effect on protein profile of *S. typhimurium* **(A)** and *S. sonnei* **(B)** at 0, 30, 60, and 150 min*.* The content of bacterial cells was separated on 12.5% SDS-PAGE after boiling for 7 min, stained with Coomassie brilliant blue and destained. Panels A and B represent individual gels, which were not modified in any way.

**
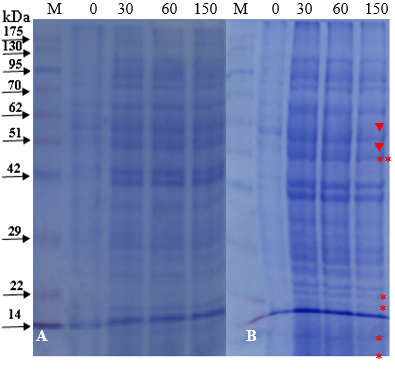
**

**Figure S3**. 12.5% reducing SDS-PAGE for effect of carbenicillin combined with camel lactoferrin on protein profile of *S. typhimurium* **(A)** and *S. sonnei* **(B)** at 0, 30, 60, and 150 min*.* The content of bacterial cells was separated on 12.5% SDS-PAGE after boiling for 7 min, stained with Coomassie brilliant blue and destained. Panels A and B represent individual gels, which were not modified in any way.

**
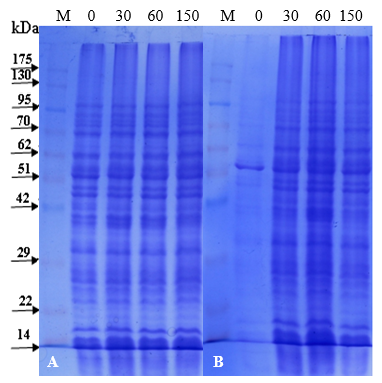
**

**Figure S4**. 12.5% reducing SDS-PAGE for chloramphenicol effect on protein profile of *S. typhimurium* **(A)** and *S. sonnei* **(B)** at 0, 30, 60, and 150 min*.* The content of bacterial cells was separated on 12.5% SDS-PAGE after boiling for 7 min, stained with Coomassie brilliant blue and destained. Panels A and B represent individual gels, which were not modified in any way.

**
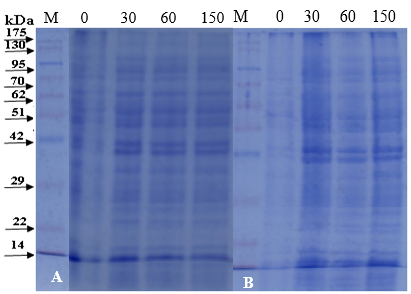
**

**Figure S5.** 12.5% reducing SDS-PAGE for effect of chloramphenicol combined with camel lactoferrin on protein profile of *S. typhimurium* **(A)** and *S. sonnei* **(B)** at 0, 30, 60, and 150 min*.* The content of bacterial cells was separated on 12.5% SDS-PAGE after boiling for 7 min, stained with Coomassie brilliant blue and destained. Panels A and B represent individual gels, which were not modified in any way.

**
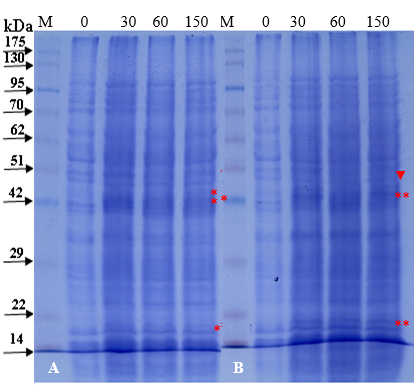
**

**Figure S6**. 12.5% reducing SDS-PAGE for imipenem effect on protein profile of *S. typhimurium* **(A)** and *S. sonnei* **(B)** at 0, 30, 60, and 150 min*.* The content of bacterial cells was separated on 12.5% SDS-PAGE after boiling for 7 min, stained with Coomassie brilliant blue and destained. Panels A and B represent individual gels, which were not modified in any way.

**
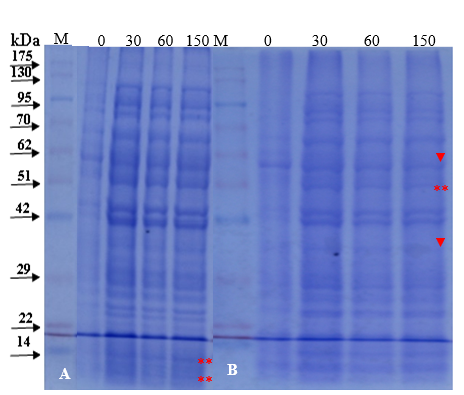
**

**Figure S7**. 12.5% reducing SDS-PAGE for effect of imipenem combined with camel lactoferrin on protein profile of *S. typhimurium* **(A)** and *S. sonnei* **(B)** at 0, 30, 60, and 150 min*.* The content of bacterial cells was separated on 12.5% SDS-PAGE after boiling for 7 min, stained with Coomassie brilliant blue and destained. Panels A and B represent individual gels, which were not modified in any way.
